# Association of Environmental Factors with Seasonal Intensity of *Erysipelothrix rhusiopathiae* Seropositivity among Arctic Caribou

**DOI:** 10.3201/eid2808.212144

**Published:** 2022-08

**Authors:** O. Alejandro Aleuy, Michele Anholt, Karin Orsel, Fabien Mavrot, Catherine A. Gagnon, Kimberlee Beckmen, Steeve D. Côté, Christine Cuyler, Andrew Dobson, Brett Elkin, Lisa-Marie Leclerc, Joëlle Taillon, Susan Kutz

**Affiliations:** University of Notre Dame, Notre Dame, Indiana, USA (O.A. Aleuy); University of Calgary, Calgary, Alberta, Canada (O.A. Aleuy, K. Orsel, F. Mavrot, S. Kutz);; One Health at UCalgary, Calgary (M. Anholt); Canada Research Chair on Northern Biodiversity, Université du Québec à Rimouski Centre of Northern Studies and Quebec Center for Biodiversity Science, Rimouski, Quebec, Canada (C.A. Gagnon);; Alaska Department of Fish and Game, Fairbanks, Alaska, USA (K. Beckmen);; Laval University, Quebec City, Quebec, Canada (S.D. Côté);; Greenland Institute of Natural Resources, Nuuk, Greenland (C. Cuyler);; Princeton University, Princeton, New Jersey, USA (A. Dobson);; Government of the Northwest Territories, Yellowknife, Northwest Territories, Canada (B. Elkin);; Government of Nunavut, Kugluktuk, Nunavut, Canada (L.-M. Leclerc);; Government of Quebec, Quebec City (J. Taillon)

**Keywords:** Erysipelothrix rhusiopathiae, Rangifer tarandus, caribou, bacteria, pathogens, serosurveillance, Arctic region

## Abstract

Several caribou (*Rangifer tarandus*) populations have been declining concurrently with increases in infectious diseases in the Arctic. *Erysipelothrix rhusiopathiae*, a zoonotic bacterium, was first described in 2015 as a notable cause of illness and death among several Arctic wildlife species. We investigated epidemiologic and environmental factors associated with the seroprevalence of *E. rhusiopathiae* in the Arctic and found that seropositivity was highest during warmer months, peaking in September, and was highest among adult males. Summer seroprevalence increases tracked with the oestrid index from the previous year, icing and snowing events, and precipitation from the same year but decreased with growing degree days in the same year. Seroprevalence of *E. rhusiopathiae* varied more during the later years of the study. Our findings provide key insights into the influence of environmental factors on disease prevalence that can be instrumental for anticipating and mitigating diseases associated with climate change among Arctic wildlife and human populations.

A 2021 report of the Intergovernmental Panel on Climate Change (*1*) reinforced that anthropogenic influences on climate systems are causing increases in temperature and extremes of weather and climate events at rates unprecedented in at least the last 2,000 years. In the Arctic, climate warming will continue at rates ≈2 times higher than those in the rest of the world, profoundly affecting biotic and abiotic systems (*1*,*2*). For example, development and death rates among pathogens and vectors and host factors such as immune response and aggregation are very sensitive to environmental conditions and extremes in climate (*3*). As a consequence, as climate change progresses and weather events become less predictable, changes in the dynamics of wildlife disease are likely to increase (e.g., changes in prevalence), directly affecting conservation biology, human health, and food safety and security in Arctic ecosystems.

*Erysipelothrix rhusiopathiae*, a gram-positive zoonotic bacterium, was first detected infecting muskoxen (*Ovibos moschatus*) in the western Canadian Arctic (*4*). During 2010–2014, a single genotype of this bacterium was associated with unusual and widespread mortality events and population declines among muskoxen in this region (*4*,*5*). During the same period, multiple genotypes of *E. rhusiopathiae* were isolated from muskoxen in Alaska, USA, and moose (*Alces americanus*) and woodland caribou (*R. tarandus caribou*) from British Columbia and Alberta, Canada, during periods of unusually high mortality for all 3 species (*6*,*7*). Recently, *E. rhusiopathiae* was identified as the cause of a disease syndrome in Pribilof Arctic foxes (*Vulpes lagopus pribilofensis*) in Alaska (*8*), and concerns have emerged regarding possible public health issues in Arctic communities (*9*). Clinical disease manifests similarly in animals and humans, including skin lesions, fever, endocarditis, and septicemia (*10*). Among domestic animals, illness from *E. rhusiopathiae* occurs under stressful circumstances, and while illness is acute, the bacteria sheds in large amounts through nasal secretions, saliva, and feces; animals with chronic infections are long-term sources of contamination (*11*). This pattern is particularly relevant because *E. rhusiopathiae* can persist for prolonged periods in the environment, including in soil and water, which are notable sources of indirect transmission (*12*). In wild systems, *E. rhusiopathiae* has been associated with individual cases, clusters, and large-scale illness events (*4*,*8*,*13*,*14*).

*Rangifer tarandus* caribou (called reindeer outside North America) are core to the structure and function of Arctic ecosystems and have profound regulatory effects on vegetation growth and diversity, as well as population dynamics among top predators (*15*). In addition, these animals are fundamental to the culture, economy, and socioeconomic wellbeing of circumpolar indigenous peoples (*16*). Several *Rangifer* populations have declined, some by 99%, in the past 15 years, with little to no evidence of recovery (*17*). Some of these declines have coincided with the emergence of pathogens and changes in the distribution, epidemiology, and effects of endemic diseases (*18*–*20*). Wildlife managers, indigenous wildlife comanagement organizations, scientists, and public health officials in the Arctic face the substantial challenge of understanding and managing the effects of emerging infectious diseases on caribou health, conservation, and food security. Determining interactions between seasonal and large-scale weather and climatic events and the dynamics of relevant pathogens is a first step towards anticipating, preparing for, and adapting to perturbations in disease ecology linked to climate changes in the Arctic (*21*).

The effects of *E. rhusiopathiae* on caribou survival and food security and on human health, along with its distribution throughout the Arctic, make it an ideal model for understanding how pathogens will be influenced by changes in environmental conditions in the future. We investigated the epidemiology of *E. rhusiopathiae* in migratory tundra caribou to quantify and report the association of environmental conditions with *E. rhusiopathiae* seropositivity in caribou. Elucidating the epidemiology of *E. rhusiopathiae* and the environmental factors influencing its seropositivity in caribou is instrumental for developing predictive frameworks to anticipate and mitigate disease risks influenced by climate change.

## Methods

### Sample Collection

We obtained frozen serum and blood on filter paper samples collected from 21 migratory tundra caribou herds during 1980–2019. Samples were collected opportunistically during capture-and-collar programs across Canada, Alaska, and Greenland. We collected information for the sampled animals on the herd name, sex, age class (immature [<24 mo of age] or adult [>24 mo of age]), pregnancy status, body condition status (lean, good, or very good) visually assessed at sampling as described elsewhere (*22*), and collection dates.

### Seroprevalence Analysis and Cutoff Determination

To determine the seroprevalence of *E. rhusiopathiae,* we used a modified ELISA (*5*). Results were expressed as percentage positivity based on a benchmark positive control; we assumed a bimodal Gaussian distribution of percentage positivity values and determined the optimal cutoffs using maximum-likelihood estimation. We calculated 95% CIs around estimated point values using bootstrapping. We classified any sample with a percentage positivity above the CI as seropositive and below the CI as seronegative. We considered serum and filter paper samples as 2 different sets and determined separate cutoffs (*5*).

### Herd-Specific Weather Conditions

 We obtained weather data from the CircumArctic Rangifer Monitoring and Assessment (CARMA) network’s caribou range climate database (https://carma.caff.is) (*23*). This dataset includes 26 variables describing daily environmental conditions in each of the seasonal territorial ranges of 22 caribou (reindeer) herds across North America, Greenland, and Eurasia; these data enabled us to calculate monthly mean residuals specific to the seasonal range used by each herd during the study period (1980–2015). As dates for caribou seasonal ranges, we used September 1–November 30 for fall range, December 1–March 31 for winter range, April 1–May 31 for spring range, June 1–30 for calving range, and July 1–August 31 for summer range. We conducted a literature review to determine weather and climatic events affecting the performance of caribou herds (Appendix 1 Table).

### Data Analysis

We used the entire dataset to calculate descriptive seroprevalence and binomial proportion 95% CIs for each caribou herd for sex, age class, and body condition class. We investigated associations among these variables and seroprevalence using a generalized linear mixed model (GLMM) with binomial distribution using herd and year of sample collection as nested random effects to address the uneven distribution of samples.

The Western Arctic, Central Arctic, and Teshekpuk Lake herds in Alaska and the Alaska–Canada transboundary Porcupine herd provided relatively rich data with samples taken across most months and over several decades; thus, we focused analyses on data from these herds. We investigated monthly distribution of *E. rhusiopathiae* seroprevalence using a GLMM with binomial distribution. We included month, age class, and sex as independent variables in the models. To account for the nonlinearity of seasonal trends, we included different polynomial degrees of the variable month in the model. We fitted models with different combinations of these independent variables and then compared models using the Akaike information criterion (AIC) (*24*).

We investigated the association between seropositivity of *E. rhusiopathiae* and weather and environmental factors using GLMM with binomial distribution. The dependent variable was seropositivity of *E. rhusiopathiae* in individual caribou during June, July, August, and September, the months with highest seroprevalence. Month of sampling was included as a random effect in the model. We obtained the independent variables from the CARMA database using temporal and spatial scales specific to each herd including effective growing degree days above 5°C (GDD5) (used to estimate growth and development of plants and insects), daily total surface precipitation, and oestrid index (as a proxy for insect harassment) from the calving range and current and previous year’s summer ranges. We included those variables in the model as the residuals of their mean values for the period under study. In addition, we pooled variables pertaining to snowing and icing events from the fall, winter, and spring ranges. We performed a separate analysis to transform correlated variables into uncorrelated principal components for snowing and icing events (*25*). We decided the number of principal components to be used as final variables on the basis of a sharp decline in consecutive eigenvalues and eigenvalues >1.0 (*26*), which identified 2 principal components describing snowing events, PCsnow1 and PCsnow2, and 2 describing icing events, PCice1 and PCice2 (Table 1; Appendix 2 Table 1). We compared models that included different combinations of fixed effects, which were not highly correlated (*r*<0.7), and interactions based on AIC and analysis of variance (ANOVA).

**Table 1:** Components used as climate indices to characterize snowing and icing events during the fall, winter, and spring seasonal ranges in the caribou territorial ranges of 4 Western Arctic herds during 1985–2014*

Event	Variable name	Description of component
Snowing events	PCsnow1	High snow depth and snow density in the fall, winter, and spring seasonal ranges and large proportion of surface area of total geographic range covered by snow in the fall
	PCsnow1	Low snow melt rate in spring and fall seasonal ranges, high snow depth and large proportion of surface area of total geographic range covered by snow in the spring.
Icing events	PCice1	High number of days with freeze/thaw events and rain on snow in fall, winter, and spring seasonal ranges.
	PCice2	High number of days with freeze/thaw events and rain during snow events in the fall seasonal range, but low in the winter and spring.

*Three herds were in Alaska (Western Arctic, Central Arctic, and Teshekpuk Lake) and 1 Alaska–Canada transboundary (Porcupine), Seasonal ranges: fall, September 1–November 30; winter, December 1–March 31; spring, April 1–May 31; calving, June 1–30; and summer, July 1–August 31. PC, principal component.

To investigate trends and variability of *E. rhusiopathiae* seroprevalence during the study period, we calculated the monthly residuals of mean seroprevalence; we used data from the 4 herds in Alaska and only from months with >8 samples. After dividing the 30-year study period into 10 groups of 3 years each, we combined 3-year totals for each month to increase monthly sample sizes. We obtained monthly residuals by calculating the absolute monthly seroprevalence over the entire study period and then subtracting it from monthly prevalence in each of these 10 periods. We quantified seroprevalence of *E. rhusiopathiae* as the proportion of seropositive samples within each period.

## Results

**Figure 1:**
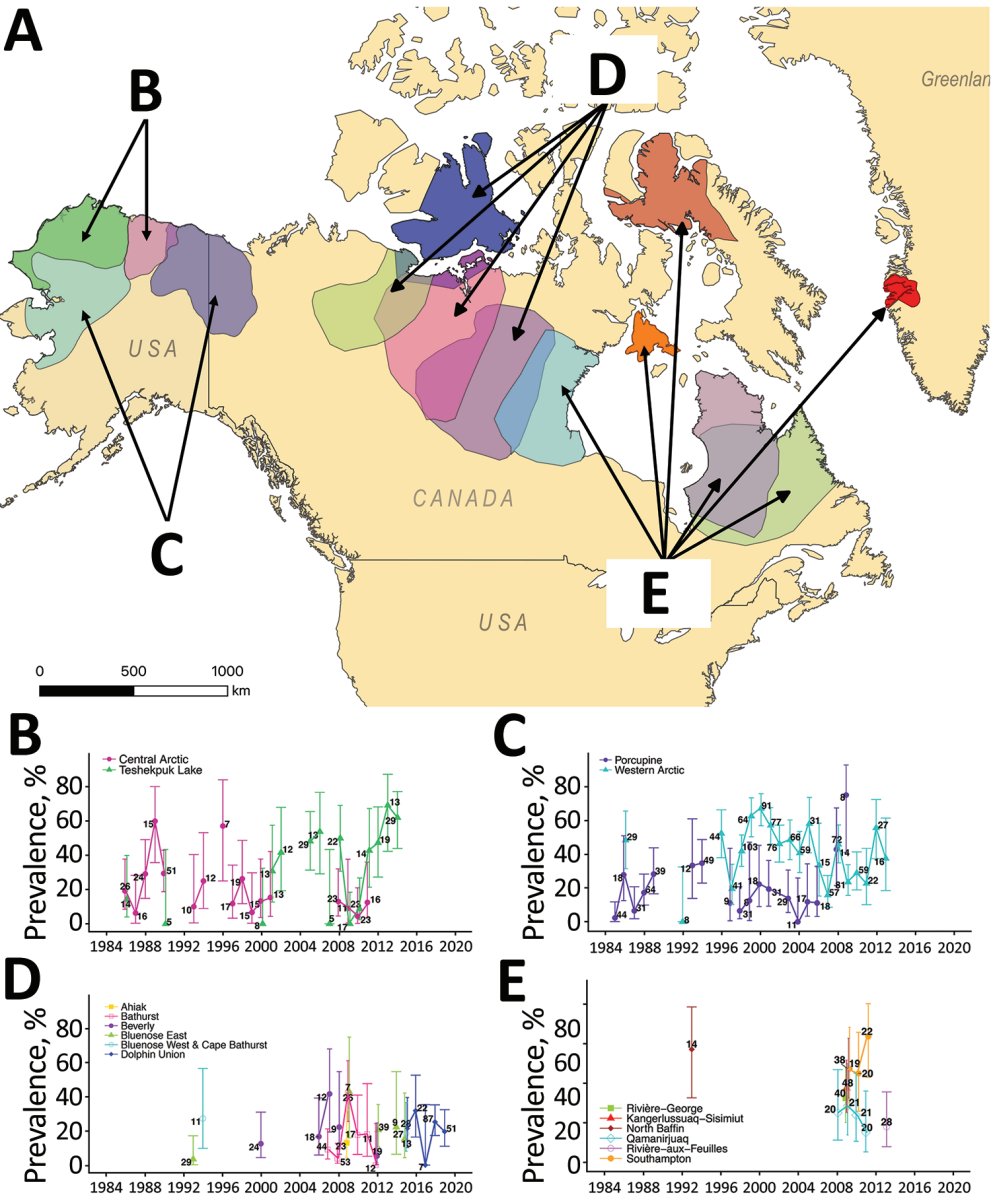
Yearly seroprevalence of *Erysipelothrix rhusiopathiae* in caribou herds with territorial ranges in B) Alaska, USA; C) Alaska and Yukon, Canada; D) north central Canada; and E) northeastern Canada, Baffin Island, Canada, and Greenland during 1980–2019. Line colors in graphs B–E correspond to colors of territorial ranges on map of sampled herds. Numbers indicate the sample size for each year; error bars indicate 95% CIs.

We analyzed 3,170 caribou samples, then randomly selected and removed duplicate samples from 125 animals sampled in >1 period, leaving 3,045 test results for the analysis. Three Alaska herds (Western Arctic, Central Arctic, and Teshekpuk Lake) and the transboundary (Alaska–Canada) Porcupine herd provided 68.4% of the samples. Seropositivity was found in 18/19 herds included in the study. In the herd with no positives (Boothia Peninsula, Nunavut, Canada), only 4 samples were analyzed. Overall, 31.4% (95% CI 29.6%–33.1%) of the samples analyzed were seropositive (Figure 1; Appendix 2 Table 2).

### Effects of Individual Traits

In the best model for investigating the association of age class and sex with *E. rhusiopathiae* seroprevalence, male caribou had a significantly higher seroprevalence than female caribou (odds ratio [OR] 1.4, 95% CI 1.1–1.8). This same model indicated that, for the age class variable, adult caribou had higher *E. rhusiopathiae* seroprevalence, but the effect was small (OR 0.7, 95% CI 0.5–1.0) (Appendix 2 Table 3). We observed no overall association between caribou body condition class and seroprevalence (n = 249) (Appendix 2 Table 4), although in winter we observed a trend in which seroprevalence in animals in poor body condition was 2 times that of animals in good body condition (χ^2^_(1,70)_ = 1.8; p = 0.2) (Appendix 2 Table 3). Pregnancy was not associated with seroprevalence.

### Seasonal Distribution of Seroprevalence

**Figure 2:**
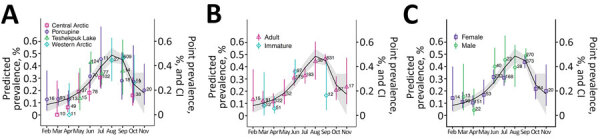
Monthly seroprevalence and predicted prevalence of *Erysipelothrix rhusiopathiae* in caribou from western North America during 1980–2019. A) By herd; B) by age; C) by sex. The predicted prevalence was determined using generalized linear models with binomial distribution using month of collection as an independent variable. We included only months with >8 samples. Error bars indicate 95% CIs.

In the 4 herds from western North America (Western Arctic, Central Arctic, Teshekpuk Lake, and Porcupine), we observed a clear seasonal pattern of higher seroprevalence during warmer months (June–September). Seasonal seroprevalence varied widely, showing a significant increase from 9.8% (binomial confidence interval [BCI] 6.2%–15.2%) in April to 32.7% (BCI 27.4%–38.5%) in June (GLMM, June vs. April: b = 1.42, SE 0.28, z = 5.1; p<0.01), reaching a peak of 45.9% seroprevalence (BCI 42.1%–49.75%) in September and significantly decreasing to 20.6% (BCI 12.7%–31.6%) in October (GLMM, October vs. September: b = −1.14, SE 0.24, z =  −4.6; p<0.01) (Figure 2, panel A; Appendix 2 Table 5). The odds for *E. rhusiopathiae* seropositivity in September were >6 times higher than in February (OR 6.5, 95% CI 1.8–40.9), March (OR 6.3, 95% CI 3.7–11.2), or April (OR 6.7, 95% CI 4.2–11.4). Including sex or age class did not improve model fit (Figure 2, panels B, C; Appendix 2 Table 6).

### Climatic and Environmental Factors Influencing Seropositivity

**Figure 3:**
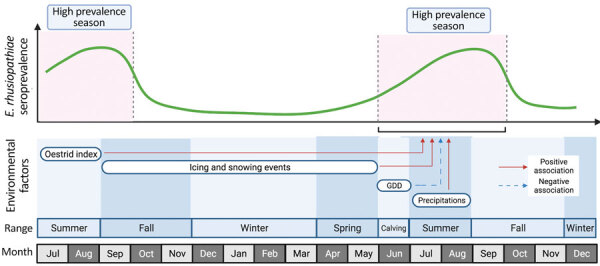
Schematic to explain the influence of environmental factors on the intensity of the seasonal prevalence of *Erysipelothrix rhusiopathiae* in caribou from Western North America during 1980–2019. Rows in the lower part of the figure indicate the temporal (month) and spatial (range) occurrence of each environmental factor. GDD, effective growing degree days (used to estimate growth and development of plants and insects).

Seropositivity of *E. rhusiopathiae* was associated with weather and environmental conditions during different seasonal ranges (Table 2; Figure 3). Including month of sample collection as a random effect significantly improved model fit (ΔAIC 11.3, ANOVA; p<0.001) (Appendix 2 Table 7). An increase in GDD5 during calving season was negatively associated with seropositivity of *E. rhusiopathiae* (OR 0.9, 95% CI 0.8–1.0). Icing events occurring during the entire length of the cold season (i.e., in fall, winter, and spring), significantly increased the chances of seropositivity for *E. rhusiopathiae* the following summer (PCice1 OR 1.13, 95% CI 1.0–1.3). More important, icing events occurring only during the fall range were enough to cause a similar increase in seropositivity the following summer (PCice2 OR 1.3, 95% CI 1.1–1.5). Summer conditions, including the surface precipitation from the same year and oestrid harassment from the previous summer, increased seropositivity of *E. rhusiopathiae* (surface precipitation OR 1.2, 95% CI 1.1–1.4; oestrid index OR 1.3, 95% CI 1.2–1.5) (Table 2; Figure 3).

**Table 2:** Estimates of the final model to investigate the association between seroprevalence of *Erysipelothrix rhusiopathiae* in caribou and herd-specific environmental conditions*

Environmental condition	Estimate (SE)	p value
Summer surface precipitation	0.19 (0.061)	0.002
Previous summer oestrid index	0.27 (0.058)	<0.001
Calving GDD5	−0.15 (0.068)	0.027
PCsnow2†	0.18 (0.066)	0.006
PCice1†	0.12 (0.059)	0.034
PCice2†	0.25 (0.066)	<0.001

*All results were significant (p<0.05). GDD5, effective growing degree days above 5°C (used to estimate growth and development of plants and insects); PC, principal component. †Described in Table 1..

### Long-Term Trends in Seroprevalence

**Figure 4:**
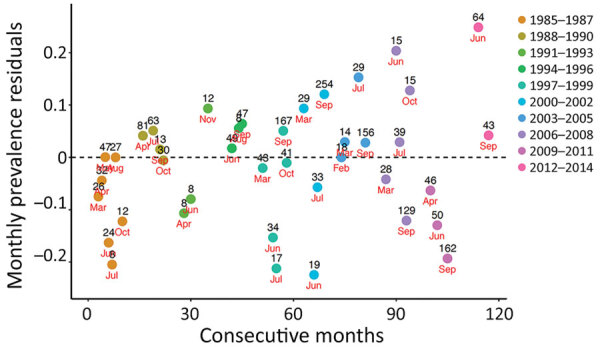
Residuals of monthly prevalence of *Erysipelothrix rhusiopathiae* in caribou from Western North America during ten 3-year time periods, 1985–2014. Only months (red text) with >8 samples (black text) were included. The residuals were calculated using 3 herds from Alaska (Western Arctic, Central Arctic, and Teshekpuk Lake) and a transboundary Alaska–Canada herd (Porcupine).

The variability of *E. rhusiopathiae* seroprevalence residuals in western North America trended upward during 1985–2014. In the first part of this period, the residuals were mostly negative with positive values that were close to 0. Conversely, during the second half of the period, the range between positive and negative residuals gradually increased, leading to more variability in seroprevalence. The 4 highest residuals occurred during the second half of the study period (Figure 4).

## Discussion

Drawing on a large repository of samples, we demonstrated that *E. rhusiopathiae* is widely distributed among North American tundra caribou herds with seroprevalence varying over space and time. We detected a seasonal pattern of higher seroprevalence during summer months; the amplitude of this seasonal pattern was associated with various environmental variables that are known stressors for caribou. Finally, the variability in seroprevalence of *E. rhusiopathiae* appeared to increase during the later years of the study period. Although the data we used for analyses originated from a complex array of sampling protocols, resulting in an unbalanced dataset, the sheer volume of samples across space and time enabled insights into factors that might influence seropositivity. Our study provides key insights into the influence of environmental factors on seroprevalence, which is instrumental for anticipating and mitigating disease-related risks from climate change for wildlife and human populations in the Arctic.

 Seropositivity to *E. rhusiopathiae* among caribou was highly seasonal, peaking during the warmest months (Figure 3). Given a ≈2-week delay after exposure before seropositivity would be detected (*27*), *E. rhusiopathiae* transmission among caribou is likely heavily influenced by seasonal factors occurring before and during calving season and early summer. The life history of migratory caribou is characterized by seasonal environmental stressors and aggregation periods that may drive this summertime peak in seroprevalence. The high energy demands of long winters and spring migration (*28*), together with periparturient immunosuppression, may decrease immunocompetence and increase the susceptibility of caribou to infections (*29*). The subsequent calving period, characterized by dense aggregations in June followed by post-calving aggregations in July, results in increased contact among caribou, which is associated with heightened transmission opportunities through high exposure to caribou secretions and excretions, such as feces, urine, saliva, nasal secretions, and placenta. These heightened transmission opportunities, together with the negative influences on overall caribou health from preceding winter environmental conditions, seasonal migration, pregnancy, and other disease issues, might be the trigger for the increased circulation of *E. rhusiopathiae* in the summer.

The amplitude of the seasonal increase in seropositivity of *E. rhusiopathiae* in a given year was influenced by weather and environmental factors in the previous year that are known to cause substantial distress in caribou: oestrid harassment and icing and snowing events (Figure 3; Appendix 2 Table 1). Insect harassment, particularly from oestrid flies, warbles (*Hypoderma tarandi*) and nose bots (*Cephenemyia trompe*), because of the increased time spent avoiding insect harassment negatively affects food intake among caribou (*30*–*33*). During the summer, warble flies lay eggs on the hair of caribou, which then hatch into larvae that penetrate the skin. Larvae migrate to the subcutaneous region on the animal’s back where they remain as third instars until the following summer when they depart their host through breathing holes in the caribou’s skin, then pupate in the environment (*34*). The migration and growth of larvae in the caribou are energetically costly. At the same time, the parasitic larvae release enzymes, serine proteases, that down-regulate host immune function, negatively influencing immune response to other pathogens (*35*), such as *E. rhusiopathiae*. Finally, the lesions in the skin left by emergent larvae may provide *Erysipelothrix* entry points, mostly because of flying insects that can act as fomites (*36*). Similarly, icing and snowing events can also negatively affect caribou performance, including body condition and pregnancy rates, and cause mass die-offs and declines in herd populations (*37*–*41*). Conversely, conditions supporting good vegetation growth, which we estimated using GDD5 as a proxy, decreased the likelihood of elevated *E. rhusiopathiae* seropositivity in the same year potentially by positively influencing intrinsic caribou health factors, such as body mass and thus, likely pathogen resistance (*40*,*42*).

Different theoretical and disease-specific approaches have demonstrated that climate variability and extreme weather events likely affect disease dynamics in hard-to-predict ways (*43*). Our study results indicating an increasing trend in the variability of *E. rhusiopathiae* seroprevalence are consistent with this dynamic and might result from the increasing variability of the Arctic climate during the study period. Environmental drivers can alter disease transmission and manifestations through direct influences on the development, persistence and mortality of pathogens, as well as by influencing the physiologic and behavioral responses of both hosts and vectors. An increase in seroprevalence in the Arctic might suggest a negative impact on caribou populations as this bacterium has been implicated in several caribou deaths in western Canada and on Arctic islands (*6*,*7*). Further understanding how weather and climate variability interacts with hosts, pathogens, and vectors to influence the epidemiology and ecology of *E. rhusiopathiae* would offer essential insights into how this host-pathogen relationship works, when measures to mitigate infections should be applied, and how disease risk for humans and wildlife will respond to anthropogenic climate change.

Determining how *E. rhusiopathiae* is maintained at high latitudes between summer peak seasons, fall, winter, and spring, is critical to understanding the seasonal dynamics of *E. rhusiopathiae*; animal reservoirs play roles in other wild systems (*10*). Close to a hundred species of birds and mammals are susceptible to *E. rhusiopathiae,* including a variety of high-latitude species (*5*,*6*,*8*). Wild rodents are a well-known host for the bacterium (*10*). Because *E. rhusiopathiae* can survive in the environment for long periods (*10*), reservoir species such as rodents that overwinter in the subnivean environment, where temperatures are milder, more stable, and perhaps more conducive for pathogen survival, might play an important role in its persistence in the extreme Arctic environment. Lemmings (e.g., *Dicrostonyx* spp., *Lemmus trimucronatus*) and voles (e.g., *Clethrionomys rutilus*, *Microtus oeconomus*) in the Arctic, display strong subnivean activity with seasonal increases in population density during winter months and profound interannual variation in population size (*44*). Another hypothesis to explain the overwinter persistence of pathogens involves migratory wild water birds, which are notable carriers of poultry pathogens like Newcastle and avian influenza viruses (*45*,*46*), meaning *E. rhusiopathiae* is not the lone exception (*47*).

We have documented the seasonality, ecology, and historical trends of *E. rhusiopathiae*, an emerging pathogen in the Arctic. Our work highlights the role of environmental factors on the seroprevalence of this zoonotic pathogen, which is infecting a key Arctic ungulate in one of the regions most affected by anthropogenic climate change. Changes in the dynamics of pathogens from the Arctic have already been documented and are expected to increasingly affect human health, food security, and wildlife conservation (*48*–*50*). Environmental conditions can affect the physiology and behaviors of caribou and have both proximate and remote consequential influences on the transmission of infectious disease pathogens such as *E. rhusiopathiae*. This information is instrumental for developing predictive frameworks to anticipate and mitigate climate change–related disease risks. For example, intensifying passive and active caribou surveillance efforts, and strengthening public health campaigns to educate persons who might be exposed (e.g., from hunted animals) on safe practices to avoid *Erysipelothrix* infections, especially in years preceded by summer seasons with a high oestrid index. Enacting efforts to mitigate the effects of emerging climate change–related disease threats offer direct benefits for developing adaptations to public health, food security, and conservation efforts.

Appendix 1 Literature review for study of variability of *Erysipelothrix rhusiopathiae* seroprevalence in Arctic caribou.

Appendix 2 Additional information on study of variability of *Erysipelothrix rhusiopathiae* seroprevalence in Arctic caribou.
