# Supplementary material for: Association of Environmental Factors with Seasonal Intensity of Erysipelothrix rhusiopathiae Seropositivity among Arctic Caribou
Source: Emerg Infect Dis. 2022 Aug;28(8):1650–8. doi: 10.3201/eid2808.212144 (PMC9328914; doi:10.3201/eid2808.212144)
Supplement: Appendix 1 — Literature review for study of variability of Erysipelothrix rhusiopathiae seroprevalence in Arctic caribou. [file 21-2144-Techapp-s1.pdf]

Article DOI: https://doi.org/10.3201/eid2808.212144

Association of Environmental Factors with Seasonal Intensity of *Erysipelothrix rhusiopathiae* Seropositivity among Arctic Caribou

Appendix 1

Literature Review

The search terms and associated strings for this review were developed and optimized through discussions with caribou and wildlife disease experts. The search was performed in English using the bibliographic database ISI Web of Science and the following search engine (caribou OR rangifer) AND (weather OR climate OR environment OR insects OR temperature OR rain OR snow OR precipitation OR ice). Among the 146 articles returned from the search, only 28 were deemed relevant at the title and abstract revision (Appendix 1 Table). These articles were reviewed and the weather/climatic events and environmental variables influencing caribou performance were summarized.

Appendix 1 Table: Summary of literature review investigating the environmental variables influencing caribou performance

Range	Environmental variables	Consequences	References
Winter	Temperature, humidity, cloudiness, precipitation	Associated with herd decline, changes in migration patterns (fall and spring), negatively associated with pregnancy rate, mass mortality,	(*1*,*2*)
	Snowfall, precipitations, cumulative snow on the ground, snow deep, snow density, rain on snow, hardness of snow, snow melting	Associated with herd decline, changes in migration patterns (fall and spring), negatively associated with pregnancy rate, mass mortality	(*1*–*11*)
	Icing events, freeze-thaw events, the hardness of snow, snow crust	Associated with body condition next fall, population trends Degree days in May positively associated with body condition next Fall	(*7*,*9*,*12*–*14*)
Spring	Snow deepness and hardness, Snow water equivalent, Soft melting snow	Influence on caribou movement (e.g., slowed walk), delayed migration departure, associated with body condition	(*2*,*4*,*12*,*15*)
	Precipitations, warm temperatures, cold temperatures, growing degree days	Changes in migration patterns (e.g., departure, arrival), associated with body condition in fall	(*2*,*12*)
Calving	Normalized difference vegetation index (NDVI), the start of plant growing season	Associated with birth mass, peak calving date	(*11*,*16*)
	Start of plant growing season	Associated with peak calving date. Delayed peak calving date significantly decreases the birth rate.	(*16*)
	Insect density (mosquitos and oestrids)	Changes in the use of the landscape (e.g., use higher altitudes, use snow patches), changes in behavior (e.g., time spent in feeding, standing, running), changes in group sizes.	(*17*)
	Blizzard	Behavioral changes (e.g., increase in bedding, posterior increasing in feeding)	(*18*)

Range	Environmental variables	Consequences	References
Summer	Oestrid flies and mosquitoes	Changes in activity budget (e.g., standing time, feeding time), negative influence on body condition, variation of group size, negative associated with calves weight in autumn	(*12,19–26*)
	Temperature, wind speed, humidity, and cloud cover	Associated with changes in activity budget (e.g., standing time, feeding time), group size, the distance among individuals, herd decline, herd increase	(*1,19*)
	Normalized difference vegetation index (NDVI)	Negative association with population growth	(*27*)
	Intensity of Arctic Oscillation (Warmer temperatures and longer growing seasons)	Positively associated with population growth, increase in dissectible fat	(*28*)
Fall	NDVI in October, snow water equivalent, warmer temperatures	Changes in fall migration patterns	(*2*)

References

1. Joly K, Klein DR, Verbyla DL, Rupp TS, Chapin FS III. Linkages between large-scale climate patterns and the dynamics of Arctic caribou populations. Ecography. 2011;34:345–52. https://doi.org/10.1111/j.1600-0587.2010.06377.x

2. Le Corre M, Dussault C, Côté SD. Weather conditions and variation in timing of spring and fall migrations of migratory caribou. J Mammal. 2016;98:260–71. https://doi.org/10.1093/jmammal/gyw177

3. Ferguson SH, Mahoney SP. The relationship between weather and caribou productivity for the La-Poile Caribou Herd, Newfoundland. Rangifer. 1991;11:151–6. https://doi.org/10.7557/2.11.4.1005

4. Adamczewski JZ, Gates CC, Soutar BM, Hudson RJ. Limiting effects of snow seasonal habitat use and diets of caribou (*Rangifer tarandus groenlandicus*) on Coats Island, Northwest Territories, Canada. Can J Zool. 1988;66:1986–96. https://doi.org/10.1139/z88-291

5. Larter NC, Nagy JA. Variation between snow conditions at Peary caribou and muskox feeding sites and elsewhere in foraging habitats on banks island in the Canadian High Arctic. Arct Antarct Alp Res. 2001;33:123–30. https://doi.org/10.1080/15230430.2001.12003414

6. Gunn A, Dragon J. Peary caribou and muskox abundance and distribution on the western Queen Elizabeth Islands, Northwest Territories and Nunavut, June-July 1997. Department of Resources, Wildlife and Economic Development, Government of the Northwest Territories; 2002.

7. Langlois A, Johnson C-A, Montpetit B, Royer A, Blukacz-Richards EA, Neave E, et al. Detection of rain-on-snow (ROS) events and ice layer formation using passive microwave radiometry: A context for Peary caribou habitat in the Canadian Arctic. Remote Sens Environ. 2017;189:84–95. https://doi.org/10.1016/j.rse.2016.11.006

8. Hansen BB, Aanes R, Herfindal I, Kohler J, Saether B-E. Climate, icing, and wild arctic reindeer: past relationships and future prospects. Ecology. 2011;92:1917–23. PubMed https://doi.org/10.1890/11-0095.1

9. Tyler NJC. Climate, snow, ice, crashes, and declines in populations of reindeer and caribou (Rangifer tarandus L.). Ecol Monogr. 2010;80:197–219. https://doi.org/10.1890/09-1070.1

10. Whitfield PH, Russell D. Recent changes in seasonal variations of climate within the range of northern caribou populations. Rangifer. 2005;(Sp. Iss. 16):11–8.

11. Couturier S, Cote SD, Otto RD, Weladji RB, Huot J. Variation in calf body mass in migratory caribou: the role of habitat, climate and movements. J Mammal. 2009;90:442–52. https://doi.org/10.1644/07-MAMM-A-279.1

12. Gagnon CA, Hamel S, Russell DE, Powell T, Andre J, Svoboda MY, et al. Merging indigenous and scientific knowledge links climate with the growth of a large migratory caribou population. J Appl Ecol. 2020;57:1644–55.

13. Collins WB, Smith TS. Effects of wind-hardened snow on foraging by reindeer (*Rangifer tarandus*). Arctic. 1991;44:217–22. https://doi.org/10.14430/arctic1541

14. Fancy SG, White RG. Energy expenditures by caribou while cratering in snow. J Wildl Manage. 1985;49:987–93. https://doi.org/10.2307/3801384

15. Duquette LS. Snow characteristics along caribou trails and within feeding areas during spring migration. Arctic. 1988;41:143–4. https://doi.org/10.14430/arctic1706

16. Chen W, Adamczewski JZ, White L, Croft B, Gunn A, Football A, et al. Impacts of climate-driven habitat change on the peak calving date of the Bathurst caribou in Arctic Canada. Polar Biol. 2018;41:953–67. https://doi.org/10.1007/s00300-018-2259-8

17. Downes CM, Theberge JB, Smith SM. The influence of insects on the distribution, microhabitat choice, and behaviour of the Burwash caribou herd. Can J Zool. 1986;64:622–9. https://doi.org/10.1139/z86-092

18. de Vos A. Behavior of barren ground caribou on their calving grounds. J Wildl Manage. 1960;24:250–8. https://doi.org/10.2307/3797511

19. Mörschel FM, Klein DR. Effects of weather and parasitic insects on behavior and group dynamics of caribou of the Delta Herd, Alaska. Can J Zool. 1997;75:1659–70. https://doi.org/10.1139/z97-793

20. Toupin B, Huot J, Manseau M. Effect of insect harassment on the behaviour of the Riviere George Caribou. Arctic. 1996;49:375–82. https://doi.org/10.14430/arctic1213

21. Russel DE, Nixon WA. Activity budgets food habitats and habitat selection of the Porcupine caribou herd during the summer insect season. In: Fifth International Reindeer/Caribou Symposium, Arvidsjaur, Sweden, August 18–22, 1988 Rangifer. 1990. p. 255–6.

22. Witter LA, Johnson CJ, Croft B, Gunn A, Gillingham MP. Behavioural trade-offs in response to external stimuli: time allocation of an Arctic ungulate during varying intensities of harassment by parasitic flies. J Anim Ecol. 2012;81:284–95. PubMed https://doi.org/10.1111/j.1365-2656.2011.01905.x

23. Weladji RB, Holand O, Almoy T. Use of climatic data to assess the effect of insect harassment on the autumn weight of reindeer (Rangifer tarandus) calves. J Zool (Lond). 2003;260:79–85. https://doi.org/10.1017/S0952836903003510

24. Hagemoen RIM, Reimers E. Reindeer summer activity pattern in relation to weather and insect harassment. J Anim Ecol. 2002;71:883–92. https://doi.org/10.1046/j.1365-2656.2002.00654.x

25. Walsh NE, Fancy SG, Mccabe TR, Pank LF. Habitat use by the Porcupine caribou herd during predicted insect harassment. J Wildl Manage. 1992;56:465–73. https://doi.org/10.2307/3808860

26. Helle T, Tarvainen L. Effects of insect harassment on weight gain and survival in reindeer *Rangifer tarandus* calves. Rangifer. 1984;4:24–7. https://doi.org/10.7557/2.4.1.488

27. Fauchald P, Park T, Tømmervik H, Myneni R, Hausner VH. Arctic greening from warming promotes declines in caribou populations. Sci Adv. 2017;3:e1601365. PubMed https://doi.org/10.1126/sciadv.1601365

28. Mallory CD, Campbell MW, Boyce MS. Climate influences body condition and synchrony of barren-ground caribou abundance in Northern Canada. Polar Biol. 2018;41:855–64. https://doi.org/10.1007/s00300-017-2248-3
