# Supplementary material for: Association of Environmental Factors with Seasonal Intensity of Erysipelothrix rhusiopathiae Seropositivity among Arctic Caribou
Source: Emerg Infect Dis. 2022 Aug;28(8):1650–8. doi: 10.3201/eid2808.212144 (PMC9328914; doi:10.3201/eid2808.212144)
Supplement: Appendix 2 — Additional information on study of variability of Erysipelothrix rhusiopathiae seroprevalence in Arctic caribou. [file 21-2144-Techapp-s2.pdf]

Article DOI: https://doi.org/10.3201/eid2808.212144

Association of Environmental Factors with Seasonal Intensity of *Erysipelothrix rhusiopathiae* Seropositivity among Arctic Caribou

Appendix 2

Appendix 2 Table 1. Principal components eigenvectors from principal component analyses on variables representing snow and icing events in the caribou range of three Alaskan herds (Western Arctic, Central Arctic, and Teshekpuk Lake), and a transboundary Alaska-Canada herd (Porcupine), between 1985–2014. These PCA included weather conditions occurring during the fall, winter, and spring

Event	Season	Variables	Principal component.1	Principal component 2
Snow	Winter	Snow depth	0.411	
		Snow density	0.464	
	Spring	Snow depth		0.676
		Snow density	0.303	
		Snowmelt rate	0.197	–0.41
		Surface snowfall		0.562
	Fall	Snow depth	0.355	
		Snow density	0.434	0.126
		Snowmelt rate		–0.161
		Surface snowfall	0.412	
Ice	Winter	No. days freeze/thaw events	0.253	–0.41
		No. days rain on snow	0.26	–0.273
	Spring	No. days freeze/thaw events	0.411	–0.205
		No. days rain on snow	0.407	–0.132
		No. days freezing rain	0.368	–0.377
	Fall	No. days freeze/thaw events	0.305	0.525
		No. days rain on snow	0.425	0.393
		No. days freezing rain	0.352	0.352

Appendix 2 Table 2. Prevalence and 95% binomial CI of *Erysypelothrix rhusiopathiae* in caribou herds during 1981–2019.

Herd	Year	Negatives	Positives	Total	Prevalence	Lower	Upper
Ahiak	2009	20	3	23	13.0	4.5	32.1
Bathurst	2007	40	4	44	9.1	3.6	21.2
	2008	51	2	53	3.8	1.0	12.8
	2009	15	11	26	42.3	25.5	61.1
	2010	14	3	17	17.6	6.2	41.0
	2011	9	2	11	18.2	5.1	47.7
	2012	12	0	12	0.0	0.0	24.2
	2013	2	1	3	33.3	1.7	79.2
Beverly	2000	21	3	24	12.5	4.3	31.0
	2006	15	3	18	16.7	5.8	39.2
	2007	7	5	12	41.7	19.3	68.0
	2008	7	2	9	22.2	6.3	54.7
	2012	18	1	19	5.3	0.3	24.6
	2014	2	0	2	0.0	0.0	65.8
Bluenose East	1993	28	1	29	3.4	0.2	17.2
	2009	4	3	7	42.9	15.8	75.0
	2012	31	8	39	20.5	10.8	35.5
	2014	7	2	9	22.2	6.3	54.7
	2015	11	2	13	15.4	4.3	42.2
Bluenose West/ Cape Bathurst	1994	8	3	11	27.3	9.7	56.6
	2015	22	5	27	18.5	8.2	36.7

Herd	Year	Negatives	Positives	Total	Prevalence	Lower	Upper
Boothia	1993	4	0	4	0.0	0.0	49.0
Central Arctic	1980	0	1	1	100.0	5.1	100.0
	1981	1	1	2	50.0	2.6	97.4
	1982	1	0	1	0.0	0.0	94.9
	1987	15	1	16	6.3	0.3	28.3
	1988	17	7	24	29.2	14.9	49.2
	1989	6	9	15	60.0	35.7	80.2
	1993	9	1	10	10.0	0.5	40.4
	1994	9	3	12	25.0	8.9	53.2
	1998	14	5	19	26.3	11.8	48.8
	1999	14	1	15	6.7	0.3	29.8
	2000	13	2	15	13.3	3.7	37.9
	2001	11	2	13	15.4	4.3	42.2
	2008	20	3	23	13.0	4.5	32.1
	2009	10	1	11	9.1	0.5	37.7
	2010	22	1	23	4.3	0.2	21.0
	2011	14	2	16	12.5	3.5	36.0
	2013	3	1	4	25.0	1.3	69.9
Dolphin Union	1993	4	0	4	0.0	0.0	49.0
	2015	22	6	28	21.4	10.2	39.5
	2016	15	7	22	31.8	16.4	52.7
	2017	7	0	7	0.0	0.0	35.4
	2018	65	22	87	25.3	17.3	35.3
	2019	41	10	51	19.6	11.0	32.5
George River	2009	27	13	40	32.5	20.1	48.0
Kangerlussuaq-Sisimiut	2009	30	18	48	37.5	25.2	51.6
Mountain	2003	7	0	7	0.0	0.0	35.4
	2005	3	1	4	25.0	1.3	69.9
North Baffin	1993	6	8	14	57.1	32.6	78.6
Porcupine	1981	5	0	5	0.0	0.0	43.4
	1984	3	0	3	0.0	0.0	56.1
	1987	29	2	31	6.5	1.8	20.7
	1988	53	11	64	17.2	9.9	28.2
	1989	28	11	39	28.2	16.5	43.8
	1993	8	4	12	33.3	13.8	60.9
	1994	32	17	49	34.7	22.9	48.7
	1998	29	2	31	6.5	1.8	20.7
	1999	7	1	8	12.5	0.6	47.1
	2000	14	4	18	22.2	9.0	45.2
	2001	25	6	31	19.4	9.2	36.3
	2003	25	4	29	13.8	5.5	30.6
	2004	11	0	11	0.0	0.0	25.9
	2005	15	2	17	11.8	3.3	34.3
	2006	16	2	18	11.1	3.1	32.8
	2008	8	6	14	42.9	21.4	67.4
	2009	2	6	8	75.0	40.9	92.9
Qamanirjuaq	2008	15	5	20	25.0	11.2	46.9
	2009	15	6	21	28.6	13.8	50.0
	2010	16	5	21	23.8	10.6	45.1
	2011	17	3	20	15.0	5.2	36.0
Leaf River	2009	20	18	38	47.4	32.5	62.7
	2013	23	5	28	17.9	7.9	35.6
Southampton	2009	10	9	19	47.4	27.3	68.3
	2010	11	9	20	45.0	25.8	65.8
	2011	8	14	22	63.6	43.0	80.3
Teshekpuk Lake	1981	1	0	1	0.0	0.0	94.9
	2000	8	0	8	0.0	0.0	32.4
	2001	9	4	13	30.8	12.7	57.6
	2002	7	5	12	41.7	19.3	68.0
	2005	15	14	29	48.3	31.4	65.6
	2006	6	7	13	53.8	29.1	76.8
	2007	5	0	5	0.0	0.0	43.4
	2008	11	11	22	50.0	30.7	69.3
	2009	17	0	17	0.0	0.0	18.4
	2010	21	2	23	8.7	2.4	26.8

Herd	Year	Negatives	Positives	Total	Prevalence	Lower	Upper
	2011	8	6	14	42.9	21.4	67.4
	2012	10	9	19	47.4	27.3	68.3
	2013	4	9	13	69.2	42.4	87.3
	2014	11	18	29	62.1	44.0	77.3
Western Arctic	1980	1	0	1	0.0	0.0	94.9
	1981	3	1	4	25.0	1.3	69.9
	1982	3	0	3	0.0	0.0	56.1
	1993	1	0	1	0.0	0.0	94.9
	1994	0	1	1	100.0	5.1	100.0
	1998	60	43	103	41.7	32.7	51.4
	1999	24	40	64	62.5	50.3	73.3
	2000	30	61	91	67.0	56.9	75.8
	2001	33	44	77	57.1	46.0	67.6
	2002	41	35	76	46.1	35.3	57.2
	2003	34	32	66	48.5	36.8	60.3
	2004	35	24	59	40.7	29.1	53.4
	2005	13	18	31	58.1	40.8	73.6
	2006	10	5	15	33.3	15.2	58.3
	2007	48	9	57	15.8	8.5	27.4
	2008	39	33	72	45.8	34.8	57.3
	2009	62	19	81	23.5	15.6	33.8
	2010	42	17	59	28.8	18.8	41.4
	2011	17	5	22	22.7	10.1	43.4
	2012	12	15	27	55.6	37.3	72.4
	2013	10	6	16	37.5	18.5	61.4
Wolf Mountain	2009	1	1	2	50.0	2.6	97.4

Appendix 2 Table 3. Comparison among generalized mixed models fitted to determine the association between seroprevalence of *Erysipelothrix rhusiopathiae* and sex and age of caribou between 1980 and 2019 in North America and Greenland.

Models	K[a]	AIC[b]	ΔAIC[c]	logLik
Sex + Age + (1 | Year) + (1 | Herd)	5	2556.2	0	–1273.1
Sex * Age + (1 | Herd) + (1 | Year)	6	2557.3	–1.1	–1272.7
Sex + (1 | Herd) + (1 | Year)	4	2558	–1.8	–1275
Age + (1 | Herd) + (1 | Year)	4	2561.6	–5.4	–1276.8
Age + Sex + (1 | Herd)	4	2586.8	–30.6	–1289.4

Appendix 2 Table 4. Seroprevalence of *Erysipelothrix rhusiopathiae* and caribou body condition in different seasons.

Season	Body condition	Negative	Positive	Total	Prevalence	Lower 95%CI	Upper 95%CI
Winter	Thin/poor	28	15	43	34.9	22.4	49.8
	Fair	2	1	3	33.3	1.7	79.2
	Good	23	5	28	17.9	7.9	35.6
Spring	Thin/poor	12	3	15	20.0	7.0	45.2
	Fair	47	11	58	19.0	10.9	30.9
	Good	13	4	17	23.5	9.6	47.3
Summer	Good	5	6	11	54.5	28.0	78.7
Fall	Thin/poor	11	5	16	31.3	14.2	55.6
	Fair	8	3	11	27.3	9.7	56.6
	Good	30	17	47	36.2	24.0	50.5

Appendix 2 Table 5. Monthly prevalence and 95% binomial Confidence Intervals of *Erysypelothrix rhusiopathiae* in four caribou herds from North America between 1981 and 2019.

Month	Herd	Year	Negative	Positives	Total	Prevalence	Lower	Upper
February	Porcupine	2003	10	2	12	16.7	4.7	44.8
	Porcupine	2004	4	0	4	0.0	0.0	49.0
March	Central Arctic	2008	10	0	10	0.0	0.0	27.8
	Porcupine	1986	1	0	1	0.0	0.0	94.9
	Porcupine	1987	24	1	25	4.0	0.2	19.5
	Porcupine	1994	0	1	1	100.0	5.1	100.0
	Porcupine	1997	1	0	1	0.0	0.0	94.9
	Porcupine	1999	6	0	6	0.0	0.0	39.0
	Porcupine	2001	23	6	29	20.7	9.8	38.4
	Porcupine	2003	2	0	2	0.0	0.0	65.8
	Porcupine	2005	8	2	10	20.0	5.7	51.0
	Porcupine	2006	16	2	18	11.1	3.1	32.8
April	Central Arctic	1985	2	1	3	33.3	1.7	79.2

Month	Herd	Year	Negative	Positives	Total	Prevalence	Lower	Upper
	Central Arctic	2009	10	0	10	0.0	0.0	27.8
	Central Arctic	2010	20	0	20	0.0	0.0	16.1
	Central Arctic	2011	14	2	16	12.5	3.5	36.0
	Porcupine	1985	28	1	29	3.4	0.2	17.2
	Porcupine	1988	48	6	54	11.1	5.2	22.2
	Porcupine	1989	21	6	27	22.2	10.6	40.8
	Porcupine	2003	2	1	3	33.3	1.7	79.2
	Western Arctic	1982	3	0	3	0.0	0.0	56.1
	Western Arctic	1992	7	0	7	0.0	0.0	35.4
	Western Arctic	1993	1	0	1	0.0	0.0	94.9
May	Central Arctic	1981	1	1	2	50.0	2.6	97.4
	Central Arctic	1982	1	0	1	0.0	0.0	94.9
	Central Arctic	1986	15	5	20	25.0	11.2	46.9
	Central Arctic	1987	12	1	13	7.7	0.4	33.3
	Central Arctic	2013	1	0	1	0.0	0.0	94.9
	Porcupine	1981	1	0	1	0.0	0.0	94.9
	Teshekpuk Lake	1981	1	0	1	0.0	0.0	94.9
	Teshekpuk Lake	1986	12	2	14	14.3	4.0	39.9
June	Central Arctic	1985	1	0	1	0.0	0.0	94.9
	Central Arctic	1993	6	1	7	14.3	0.7	51.3
	Central Arctic	1994	9	3	12	25.0	8.9	53.2
	Central Arctic	1998	14	5	19	26.3	11.8	48.8
	Central Arctic	1999	14	1	15	6.7	0.3	29.8
	Central Arctic	2000	13	2	15	13.3	3.7	37.9
	Central Arctic	2001	3	0	3	0.0	0.0	56.1
	Central Arctic	2010	2	1	3	33.3	1.7	79.2
	Central Arctic	2013	2	1	3	33.3	1.7	79.2
	Porcupine	1984	3	0	3	0.0	0.0	56.1
	Porcupine	1985	9	0	9	0.0	0.0	29.9
	Porcupine	1986	10	4	14	28.6	11.7	54.6
	Porcupine	1988	3	4	7	57.1	25.0	84.2
	Porcupine	1994	23	14	37	37.8	24.1	53.9
	Teshekpuk Lake	1991	0	1	1	100.0	5.1	100.0
	Teshekpuk Lake	2000	1	0	1	0.0	0.0	94.9
	Teshekpuk Lake	2007	5	0	5	0.0	0.0	43.4
	Teshekpuk Lake	2008	2	8	10	80.0	49.0	94.3
	Teshekpuk Lake	2009	15	0	15	0.0	0.0	20.4
	Teshekpuk Lake	2010	17	2	19	10.5	2.9	31.4
	Teshekpuk Lake	2011	6	6	12	50.0	25.4	74.6
	Teshekpuk Lake	2012	10	9	19	47.4	27.3	68.3
	Teshekpuk Lake	2013	4	9	13	69.2	42.4	87.3
	Teshekpuk Lake	2014	11	18	29	62.1	44.0	77.3
July	Central Arctic	1987	1	0	1	0.0	0.0	94.9
	Central Arctic	1988	13	5	18	27.8	12.5	50.9
	Central Arctic	1989	6	9	15	60.0	35.7	80.2
	Central Arctic	1990	15	10	25	40.0	23.4	59.3
	Central Arctic	1993	3	0	3	0.0	0.0	56.1
	Central Arctic	1997	15	2	17	11.8	3.3	34.3
	Central Arctic	2001	8	2	10	20.0	5.7	51.0
	Central Arctic	2008	10	3	13	23.1	8.2	50.3
	Porcupine	1985	6	0	6	0.0	0.0	39.0
	Porcupine	1986	0	1	1	100.0	5.1	100.0
	Porcupine	2000	0	3	3	100.0	43.9	100.0
	Porcupine	2008	0	1	1	100.0	5.1	100.0
	Teshekpuk Lake	1990	5	0	5	0.0	0.0	43.4
	Teshekpuk Lake	2000	7	0	7	0.0	0.0	35.4
	Teshekpuk Lake	2001	9	4	13	30.8	12.7	57.6
	Teshekpuk Lake	2005	14	12	26	46.2	28.8	64.5
	Teshekpuk Lake	2006	6	7	13	53.8	29.1	76.8
	Teshekpuk Lake	2008	9	3	12	25.0	8.9	53.2
	Teshekpuk Lake	2011	1	0	1	0.0	0.0	94.9
August	Central Arctic	1996	3	4	7	57.1	25.0	84.2
	Porcupine	1981	1	0	1	0.0	0.0	94.9
	Porcupine	1994	1	0	1	0.0	0.0	94.9
	Western Arctic	1986	15	12	27	44.4	27.6	62.7
September	Central Arctic	1988	1	1	2	50.0	2.6	97.4
	Porcupine	1981	2	0	2	0.0	0.0	65.8
	Porcupine	1987	4	0	4	0.0	0.0	49.0

Month	Herd	Year	Negative	Positives	Total	Prevalence	Lower	Upper
	Porcupine	1988	1	1	2	50.0	2.6	97.4
	Porcupine	1989	5	4	9	44.4	18.9	73.3
	Porcupine	1994	1	0	1	0.0	0.0	94.9
	Teshekpuk Lake	2002	7	5	12	41.7	19.3	68.0
	Teshekpuk Lake	2009	1	0	1	0.0	0.0	94.9
	Teshekpuk Lake	2011	1	0	1	0.0	0.0	94.9
	Western Arctic	1986	0	2	2	100.0	34.2	100.0
	Western Arctic	1994	0	1	1	100.0	5.1	100.0
	Western Arctic	1995	1	0	1	0.0	0.0	94.9
	Western Arctic	1996	21	23	44	52.3	37.9	66.2
	Western Arctic	1998	1	3	4	75.0	30.1	98.7
	Western Arctic	1999	11	29	40	72.5	57.2	83.9
	Western Arctic	2000	24	49	73	67.1	55.7	76.8
	Western Arctic	2001	33	43	76	56.6	45.4	67.1
	Western Arctic	2002	4	2	6	33.3	9.7	70.0
	Western Arctic	2004	35	23	58	39.7	28.1	52.5
	Western Arctic	2005	13	18	31	58.1	40.8	73.6
	Western Arctic	2007	47	9	56	16.1	8.7	27.8
	Western Arctic	2008	39	33	72	45.8	34.8	57.3
	Western Arctic	2009	17	6	23	26.1	12.5	46.5
	Western Arctic	2010	41	17	58	29.3	19.2	42.0
	Western Arctic	2011	16	5	21	23.8	10.6	45.1
	Western Arctic	2012	12	15	27	55.6	37.3	72.4
	Western Arctic	2013	10	6	16	37.5	18.5	61.4
October	Central Arctic	1986	6	0	6	0.0	0.0	39.0
	Central Arctic	1987	2	0	2	0.0	0.0	65.8
	Central Arctic	1988	3	1	4	25.0	1.3	69.9
	Central Arctic	1990	21	5	26	19.2	8.5	37.9
	Porcupine	1981	1	0	1	0.0	0.0	94.9
	Porcupine	1986	2	0	2	0.0	0.0	65.8
	Porcupine	1987	1	1	2	50.0	2.6	97.4
	Porcupine	1994	4	2	6	33.3	9.7	70.0
	Western Arctic	1992	1	0	1	0.0	0.0	94.9
	Western Arctic	1997	2	0	2	0.0	0.0	65.8
	Western Arctic	2006	10	5	15	33.3	15.2	58.3
	Western Arctic	2010	1	0	1	0.0	0.0	94.9
November	Porcupine	1993	8	4	12	33.3	13.8	60.9
	Porcupine	1994	3	0	3	0.0	0.0	56.1
	Porcupine	1998	5	0	5	0.0	0.0	43.4

Appendix 2 Table 6. Comparison among generalized linear models fitted to determine the association between seroprevalence of *Erysipelothrix rhusiopathiae* and month of collection, sex and age of caribou between 1980 and 2019 in western North America.

No.	Models	K[a]	AIC[b]	ΔAIC[c]	logLik
1	Month + Sex + I(Month^4)+ I(Month^10)+ I(Month^11)	6	1876.2	0.00	–932.09
2	Month + I(Month^4) + I(Month^10) + I(Month^11)	5	1876.8	0.57	–933.38
3	Month*Sex + I(Month^4) + I(Month^10) + I(Month^11)	7	1878.2	2.00	–932.08
4	Month + Age + I(Month^4) + I(Month^10) + I(Month^11)	6	1878.8	2.57	–933.37
5	Month*Age + Sex + I(Month^4) + I(Month^10) + I(Month^11)	8	1879.7	3.49	–931.81
6	Month*Sex + Age + I(Month^4) + I(Month^10) + I(Month^11)	8	1880.2	3.98	–932.06
7	Month*Age + I(Month^4) + I(Month^10) + I(Month^11)	7	1880.3	4.11	–933.13
8	Month	2	1912.3	36.07	–954.14
9	Sex	2	1978.2	101.97	–987.10
10	Age	2	1991.6	115.41	–993.81

Appendix 2 Table 7. Candidate models (binomial GLM, logit link) to explain the association of seroprevalence of *Erysipelothrix rhusiopathiae* in caribou and herd specific environmental conditions*

No.	Models	K	AIC	ΔAIC	AIC Wt	Cum AIC Wt
1	Daily surface precipitation summer + Oestrid index prev. summer + Cumulative degree days calving + PC2Snow + PC1Ice + PC2Ice	8	1950.46	0	0.50	0.50
2	Daily surface precipitation summer + Oestrid index summer + Oestrid index prev. summer + Cumulative degree days calving +PC2Snow + PC1Ice +PC2Ice	9	1951.80	1.34	0.26	0.76
3	Daily surface precipitation summer + Oestrid index prev. summer + Cumulative degree days calving + PC1Snow + PC2Snow + PC1Ice + PC2Ice	10	1953.24	2.78	0.12	0.88
4	Daily surface precipitation summer + Daily surface precipitation prev. summer + Oestrid index prev. summer + Cumulative degree days calving + PC1Snow + PC2Snow + PC1Ice + PC2Ice	11	1955.08	4.62	0.05	0.93
5	Oestrid index prev. summer + Daily surface precipitation summer + PC2Snow + PC2Ice	6	1955.16	4.70	0.05	0.98
6	Daily surface precipitation summer+ Oestrid index summer + Daily surface precipitation prev. summer + Oestrid index prev. summer + Cumulative degree days calving + Oestrid index calving + PC1Snow + PC2Snow + PC1Ice + PC2Ice	12	1957.04	6.58	0.02	0.99
7	Oestrid index prev. summer + Cumulative degree days calving + PC2Snow + PC1Ice + PC2Ice	7	1960.66	10.20	0	1
8	Oestrid index prev. summer + PC2Snow + PC2Ice	5	1965.65	15.19	0	1
9	Daily surface precipitation prev. summer + Oestrid index prev. summer + Cumulative degree days calving + Oestrid index calving + PC1Snow + PC2Snow	8	1969.02	18.56	0	1
10	Oestrid index prev. summer	2	1992.54	42.08	0	1

*K, no. of parameters; AIC, Akaike information criterion; ΔAIC, AIC-min(AIC); AIC Wt, AIC weight; Cum AIC Wt, AIC cumulative weight
